# Investigation of the Effect of Pregnant Women’s Maternal Health Literacy on Healthy Lifestyle Behaviors

**DOI:** 10.3390/healthcare14131928

**Published:** 2026-07-01

**Authors:** Cengiz Şanlı, Özlem Doğan Yüksekol, Nazlı Baltacı, Mihriban Ulucan

**Affiliations:** 1 Department of Obstetrics and Gynecology, Turkish Ministry of Health Elazığ Fethi Sekin City Hospital, University of Health Sciences, Elazığ 23000, Türkiye; 2 Department of Midwifery, Faculty of Health Sciences, Yozgat Bozok University, Yozgat 66000, Türkiye; ozlem.d.yuksekol@bozok.edu.tr; 3 Department of Nursing, Faculty of Health Sciences, Ondokuz Mayıs University, Samsun 55000, Türkiye; nazli.baltaci@omu.edu.tr; 4 Department of Midwifery, Faculty of Health Sciences, Fırat University, Elazığ 23000, Türkiye; m.ulucan@firat.edu.tr

**Keywords:** maternal health literacy, healthy lifestyle behaviors, pregnancy, public health, prenatal care

## Abstract

**Highlights:**

**What are the main findings?**

- Maternal health literacy significantly predicts healthy lifestyle behaviors in pregnant women, explaining 47.4% of their variance.
- Sociodemographic factors such as education level, employment status, and prenatal care utilization are associated with healthier lifestyle behaviors during pregnancy.

**What are the implications of the main findings?**

- Routine assessment of maternal health literacy should be integrated into prenatal care services to identify women at risk of inadequate lifestyle behaviors.
- Targeted health literacy-based interventions, tailored to sociodemographic risk profiles, may improve maternal and neonatal health outcomes.

**Abstract:**

**Background/Objectives**: Health literacy (HL) plays a fundamental role in maternal and neonatal outcomes by influencing women’s ability to access, understand, and apply health information during pregnancy. This study was conducted to examine the relationship between pregnant women’s maternal health literacy levels and their healthy lifestyle behaviors. **Methods**: Data were collected using the Personal Information Form, the Maternal Health Literacy Scale in Pregnancy (MHLS-P), and the Healthy Lifestyle Behaviors Scale in Pregnant Women (HLBS-PW). **Results**: The mean MHLS-P score of the pregnant women was found to be 77.29 ± 14.85, indicating a sufficient level of maternal health literacy. The mean HLBS-PW score was 118.21 ± 14.87, indicating that the pregnant women exhibited healthy lifestyle behaviors above the moderate level. **Conclusions**: These findings emphasize the necessity of integrating systematic, health literacy-specific strategies into prenatal care based on prior health literacy assessments in order to promote informed decision-making and improve maternal and child health outcomes.

## 1. Introduction

Pregnancy is a period during which both maternal and fetal health require careful monitoring. Promoting healthy lifestyle behaviors among pregnant women is highly important for maternal and fetal health [1,2]. While positive health behaviors such as adequate and balanced nutrition, stress management, regular exercise, sufficient sleep, avoidance of smoking, and hygiene practices have been reported to have beneficial effects on maternal and fetal health, unhealthy behaviors are associated with health problems such as preterm birth, excessive weight gain, low birth weight, preeclampsia, and hypertension [3,4]. The literature indicates that factors such as age, education, socioeconomic status, the presence of social support, gestational week, cultural factors, and health literacy play an important role in shaping healthy lifestyle behaviors [2,4,5].

The World Health Organization defines the health literacy as “the skills that enable individuals to access, understand and use the health information in order to protect and maintain the health” [6]. The American Medical Association (AMA) defines health literacy as “the degree to which individuals have the capacity to obtain, understand, and comprehend the health information and services needed to make proper decisions related to health” [7]. Maternal health literacy, on the other hand, refers to a woman’s ability to participate in activities that improve both her own health and that of her child. Maternal health literacy is described as a cognitive and social skill that reflects women’s motivation and ability to appropriately access, understand, and use information to protect their own health and that of their children [8]. Maternal health literacy is one of the most cost-effective strategies for reducing maternal and infant morbidity and mortality in both the prenatal and postnatal periods. Moreover, women’s levels of maternal health literacy before, during, and after pregnancy directly influence their children’s health during the growth and developmental stages [9]. It has been reported in the 2019–2023 Strategic Plan of the Republic of Turkiye Ministry of Health that only 31.1% of the population has a high level of health literacy, and it has been targeted to “promote and disseminate healthy living” for the purpose of “creating a high health-literate society” [10]. Having a good level of health literacy enables individuals to manage their health by making informed choices, reduce health risks, improve quality of life, use community resources more efficiently, and ensure the equitable functioning of the healthcare system [11]. Pregnancy may also be considered the most appropriate period for using health services, learning health-related information, and improving health literacy [12]. Therefore, it is necessary to evaluate behavioral determinants in order to develop interventions aimed at promoting healthy lifestyles. The Theory of Planned Behavior (TPB) is based on the premise that individuals are rational and consider the consequences before engaging in certain actions [13]. According to the theory, behavioral intention, shaped by attitude toward behavior, subjective norms, and perceived behavioral control, can predict behavior. Similarly, the Knowledge, Attitudes, and Practices (KAP) model, along with other theoretical models, can be used by healthcare providers to assess the impact of interventions designed to improve maternal health literacy. The KAP model is a conceptual framework that can help determine an individual’s knowledge, attitudes, and behaviors regarding a particular issue. This model is frequently applied in the health field to analyze levels of knowledge and behavior among healthcare providers and their impact on individuals [14].

Although pregnancy is a natural process, it is a period that carries significant risks. During this time, problems arising from pregnant women’s biophysical, psychological, and socioeconomic conditions may negatively affect daily life activities and reduce self-care capacity [15]. In a study conducted by Yılmazel, more than half of the women were overweight or obese, and they had low health literacy levels [16]. In a study by Astantekin et al., individuals with higher health literacy were more likely to receive preconception counseling, attended regular health check-ups, used folic acid, and were physically active for more than three days a week [17]. In addition, another study revealed that low levels of health literacy were strongly associated with tobacco use during pregnancy [18]. An increase in women’s health literacy has been associated with an increase in healthy lifestyle behaviors. However, a review of the literature indicates that there are limited studies on the level of maternal health literacy. Therefore, it is necessary to examine the relationship between maternal health literacy and healthy lifestyle behaviors.

## 2. Materials and Methods

### 2.1. Study Design

This study used a cross-sectional and correlational design.

### 2.2. Population and Sample of the Study

The study population consisted of pregnant women who attended the Obstetrics and Gynecology Outpatient Clinic of Elazığ Fethi Sekin City Hospital. The study sample comprised pregnant women who voluntarily participated in the study and met the inclusion criteria. The study was conducted after ethics committee approval had been obtained from the Ondokuz Mayıs University Social and Human Sciences Research Ethics Committee (code: 2026-134; date of approval: 27 February 2026) and the necessary institutional permissions. The study population consisted of women who visited the relevant outpatient clinic of the hospital where the research was conducted during the data collection period. The sample size was calculated as 260 using G*power (version 3.1.9.7, Heinrich Heine University, Düsseldorf, Germany) analysis with 95% power and a 3% margin of error, and, after considering potential data loss, the study was completed with 280 participants. Research data were collected face-to-face at the hospital, primarily from pregnant women who volunteered to participate after being informed about the study. The “Personal Information Form,” the “Maternal Health Literacy Scale in Pregnancy,” and the “Healthy Lifestyle Behaviors Scale in Pregnant Women” were used to collect data. The women completed the questionnaires themselves in approximately 15 min.

### 2.3. Inclusion Criteria of the Research

1. Voluntarily consenting to participate in the study;
2. Being over the age of 18;
3. Being pregnant;
4. Being able to read and understand Turkish.

### 2.4. Exclusion Criteria of the Research

Being unwilling to participate in the study.

### 2.5. Data Collection Tools

The research data were collected using a Personal Information Form, the Maternal Health Literacy Scale in Pregnancy, and the Healthy Lifestyle Behaviors Scale in Pregnant Women.

### 2.6. Personal Information Form

The research data were collected between 1 March 2026 and 3 April 2026. This form was prepared by the researchers in accordance with the existing literature. The form consists of 18 questions covering the participants’ demographic characteristics, obstetric characteristics, and sources of health-related information.

### 2.7. Maternal Health Literacy Scale in Pregnancy (MHLS-P)

The Turkish validity and reliability study of the scale which was originally developed by Taheri et al. [19] to evaluate the maternal health literacy levels of pregnant women, was conducted by Abay et al. [20]. The scale consists of 48 items. Each item is scored on a 5-point Likert scale ranging from 1 to 5. Items 1–21, which assess Pregnancy-Related Health Information are scored as “I don’t know at all” (1), “I know very little” (2), “I know a little” (3), “I know well” (4), and “I know very well” (5). Items 22–48, which assess Functional Health Literacy are scored as “Never” (1), “Rarely” (2), “Sometimes” (3), “Often” (4), and “Always” (5). The MHLS-P consists of four subscales: (1) Maternal Health Information, (2) Seeking Maternal Health Information, (3) Evaluating Maternal Health Information and (4) Decision-Making Related to Maternal Health and Maternal Health Behavior. The total scores obtained from the MHLS-P are divided into four categories: (1) Inadequate: 0–50 points, (2) Problematic: 50.166 points, (3) Sufficient: 66.1–84, (4) Excellent: 84.6–100. The “inadequate” and “problematic” categories represent limited health literacy, while the “sufficient” and “excellent” categories define desired health literacy. In the Turkish adaptation study, the total Cronbach’s alpha reliability coefficient was reported as 0.96 [20], while in the current study, it was found to be 0.96.

### 2.8. Healthy Lifestyle Behaviors Scale in Pregnant Women (HLBS-PW)

The Healthy Lifestyle Behaviors Scale in Pregnant Women (HLBS-PW), developed by Yılmaz and Karahan [21], can be administered to all pregnant women and is completed by the participants themselves. The scale is a 5-point Likert-type instrument consisting of a total of 29 items and six subscales (pregnancy responsibility, nutrition, hygiene, physical activity, travel, acceptance of pregnancy). The scale items are scored on a numerical scale from 5 to 1, ranging from “always” to “never.” There are no reverse-scored items in the scale, and higher scores obtained from the total scale indicate more positive and higher levels of healthy lifestyle behaviors. The Cronbach’s alpha reliability coefficient for the total scale was reported as 0.83 [21], whereas in the present study, it was found to be 0.91.

### 2.9. Statistical Analysis

The data obtained from the study were analyzed using IBM SPSS Statistics version 25.0. Data on the personal characteristics of the pregnant women were presented using frequencies and percentage distributions, while continuous variables were reported as mean, standard deviation, minimum, and maximum values. The normality of the data was assessed using skewness and kurtosis values (within the range of ±2). Because the data were normally distributed, the Independent Samples *t*-test was used for comparisons between two groups, and One-Way Analysis of Variance (ANOVA) was used for comparisons involving more than two groups. The relationships between scale scores were examined using Pearson correlation analysis. The linear regression analysis was performed in order to determine the effect of maternal health literacy in pregnancy on healthy lifestyle behaviors. Multicollinearity among independent variables was assessed using Variance Inflation Factor (VIF) and tolerance statistics. Tolerance values > 0.20 and VIF values < 5 were considered indicative of the absence of multicollinearity. The level of statistical significance was set at *p* < 0.05.

Ethics Approval: This study was conducted in accordance with the ethical principles outlined in the 2013 update of the Declaration of Helsinki. Before the research began, formal permission was obtained from the Ondokuz Mayıs University Social and Human Sciences Research Ethics Committee (code: 2026-134; date of approval: 27 February 2026). The purpose of the study was explained to the pregnant women who agreed to participate, and written, signed informed consent was obtained from all participants prior to data collection.

## 3. Results

The mean age of the pregnant women was 29.33 ± 5.27 years, the mean duration of marriage was 6.04 ± 5.03 years, the mean number of pregnancies was 2.10 ± 1.13, and the mean number of children was 0.97 ± 0.99. Most of the pregnant women were higher education graduates, unemployed, lived in a nuclear family, had planned and spontaneous pregnancies, had no pregnancy-related complications, received regular prenatal care, and regularly followed pregnancy-related health information sources (Table 1).

The mean total score for the Maternal Health Literacy Scale in Pregnancy (MHLS-P) was 77.29 ± 14.85, while the mean total score for the Healthy Lifestyle Behaviors Scale in Pregnant Women (HLBS-PW) was 118.21 ± 14.87. Accordingly, it was determined that while the women had a sufficient level of health literacy, they also exhibited healthy lifestyle behaviors above the moderate level (Table 2).

Statistically significant differences were found in mean HLBS-PW scores according to the pregnant women’s educational status, employment status, regular prenatal care status, regular following of pregnancy-related health information sources, and their spouses’ educational status (respectively *p* < 0.001; *p* = 0.003; *p* = 0.031; *p* = 0.002; *p* < 0.001). Accordingly, healthy lifestyle behaviors were significantly higher among pregnant women who had higher education levels, whose spouses had higher education levels, were employed, received regular prenatal care, and regularly followed pregnancy-related health information sources (Table 3).

Weak to moderate positive significant correlations were identified between the mean scores of the HLBS-PW and all its subscales and the mean scores of the MHLS-P and all its subscales (*p* < 0.001). Accordingly, as maternal health literacy levels increased, healthy lifestyle behaviors also increased (Table 4).

In the regression analysis, in which the descriptive characteristics of pregnant women related to healthy lifestyle behaviors were evaluated together with their maternal health literacy levels, it was determined that maternal health literacy was found to have a significant effect on healthy lifestyle behaviors (R = 0.689; adjusted R^2^ = 0.474; F = 41.081; *p* < 0.001). The maternal health literacy levels of the pregnant women explained 47.4% of the variance in their healthy lifestyle behaviors. A one-unit increase in the maternal health literacy score resulted in a 0.636-unit increase in the healthy lifestyle behavior score. Furthermore, the results of the multicollinearity analysis indicated that there was no substantial multicollinearity among the independent variables (Tolerance = 0.63–0.92; VIF = 1.07–1.57) (Table 5).

## 4. Discussion

Maternal health literacy can influence pregnant women’s health awareness, help them identify risks, improve the quality of prenatal care, and contribute positively to pregnancy outcomes [22].

In this study, pregnant women were found to have an adequate level of health literacy, while their healthy lifestyle behaviors were above the moderate level (Table 2). The study conducted by Doğukan-Özkan and colleagues also reported findings consistent with adequate health literacy levels among pregnant women [23]. However, contrary to the present findings, Amiresmaili and colleagues found that most participants had inadequate or borderline health literacy [24]. The inadequate literacy levels in that study may have resulted from differences in participants’ social, cultural, and educational characteristics. Similarly, Kırca and colleagues reported that pregnant women’s total scores from the Healthy Lifestyle Behaviors Scale (HLBS) were moderate. Those findings support the results of the present study [25].

Factors such as age, socioeconomic status, education level, and employment status have been reported to affect health literacy levels among pregnant women [26,27]. In this study, women who had higher education levels, whose spouses had higher education levels, and those who were employed, received regular prenatal care, and consistently followed health information sources related to pregnancy were found to have higher healthy lifestyle behavior scores (Table 3). The study conducted by Şahin and colleagues also found that pregnant women with higher health literacy levels had greater knowledge of prenatal diagnosis and care [28]. This finding supports the potential value of incorporating routine health literacy assessment into antenatal counseling, particularly for sociodemographic subgroups identified in our study as having lower healthy lifestyle behavior scores, such as women with lower education levels or irregular prenatal care attendance. In another study, Leila and colleagues reported that the lowest health literacy scores among pregnant participants were observed in housewives (73.9%), whereas the highest scores were observed among employed pregnant women (88.2%) [29]. These findings are consistent with the literature.

In the literature, it is emphasized that improving women’s health literacy is crucial for achieving positive health outcomes for women, infants, and society, and health literacy levels play a significant role in pregnant women’s perception of prenatal risks [4,30,31]. Healthy lifestyle behaviors also influence a woman’s approach to pregnancy and are key determinants of pregnancy progression. The World Health Organization has stated that approximately three-quarters of maternal deaths in developed countries and nearly half in less developed or developing countries are due to negative health behaviors [32]. Health literacy is therefore critical in shaping pregnant women’s perception of prenatal risks [31]. Given that negative health behaviors account for a substantial proportion of maternal deaths, our findings suggest that maternal health literacy may represent a modifiable, preventable target for public health strategies aimed at reducing adverse maternal outcomes. In this study, healthy lifestyle behaviors were found to increase as maternal health literacy levels increased (Table 4). A study conducted in China with 258 pregnant women found that higher health literacy positively influenced postpartum health behaviors, self-care skills, and healthy lifestyles [33]. Similarly, a study in Iran with 323 pregnant women revealed that higher health literacy levels were associated with more positive pregnancy outcomes [34]. The research findings are consistent with the literature. The relevance of maternal informed decision-making extends beyond antenatal lifestyle behaviors to choices made throughout the broader continuum of maternity care, including decisions regarding mode of delivery. For instance, a recent five-year retrospective cohort study comparing water birth with conventional vaginal delivery demonstrated that maternal characteristics and care preferences are associated with differences in perinatal outcomes [35]. Although that study did not directly assess health literacy, it illustrates how informed maternal choices regarding the mode and setting of childbirth may meaningfully shape maternal and neonatal outcomes, reinforcing the broader argument that maternal knowledge and informed decision-making play a role not only during pregnancy but across the full continuum of maternity care.

In our study, a one-unit increase in the maternal health literacy score resulted in a 0.636-unit increase in the healthy lifestyle behavior score. This finding indicates that maternal health literacy significantly influences healthy lifestyle behaviors among pregnant women. It is noteworthy that the level of maternal health literacy among pregnant women explains 47.4% of the variance in healthy lifestyle behaviors (Table 5). This value indicates that maternal health literacy explained a substantial proportion of the variance in healthy lifestyle behaviors. However, because both constructs were measured using self-reported questionnaires administered at the same time point, common-method variance may have partially strengthened the observed associations. Therefore, the findings should be interpreted with caution. In particular, because clinical maternal and neonatal outcomes were not assessed in this study, our findings establish an association between maternal health literacy and self-reported lifestyle behaviors but do not demonstrate that improving health literacy will causally improve clinical pregnancy outcomes. Şimşek-Küçükkelepçe and colleagues reported that health literacy levels influence women’s quality of life [36].

## 5. Conclusions

This study demonstrated that pregnant women had sufficient levels of maternal health literacy and exhibited healthy lifestyle behaviors above a moderate level. A significant positive association was identified between maternal health literacy and healthy lifestyle behaviors. These findings suggest that improving maternal health literacy may improve healthy lifestyle practices during pregnancy. Integrating structured health literacy assessments and targeted educational interventions into routine prenatal care may contribute to healthier lifestyle practices during pregnancy; however, since clinical maternal and neonatal outcomes were not assessed in this study, this potential benefit remains to be confirmed by future intervention studies.

### Limitations of This Study

This study has several limitations. First, the sample predominantly consisted of women with relatively higher educational levels, planned pregnancies, and regular prenatal care attendance; consequently, women with low health literacy, unplanned pregnancies, or limited access to prenatal care—who may be at greater risk of adverse maternal and neonatal outcomes—are likely underrepresented in this study. Second, as participation was voluntary, a potential volunteer bias cannot be excluded, since women who agreed to participate may differ systematically from non-participants in terms of motivation, health awareness, and engagement with healthcare services, which may limit the generalizability of the findings. Third, the use of self-reported measurement tools may have introduced response bias. Fourth, the cross-sectional design prevents the establishment of causal relationships between maternal health literacy and healthy lifestyle behaviors. Fifth, since both maternal health literacy and healthy lifestyle behaviors were assessed using self-reported questionnaires, common-method bias may have contributed to the observed associations and may partially explain the relatively high proportion of explained variance. Finally, because the study was conducted in a single center within the Turkish healthcare system, the findings reflect the sociocultural and healthcare delivery context of Turkey, where maternal health literacy levels, prenatal care utilization patterns, and health communication practices may differ from those in other countries; therefore, the findings may not be generalizable to all pregnant populations or other cultural and healthcare contexts. Future multicenter studies including more diverse and vulnerable maternal populations are warranted to enhance the generalizability of these findings.

## Figures and Tables

**Table 1:** Distribution of descriptive characteristics of the women.

Characteristics		Mean	SD
Age (years) (min. 18–max. 43)		29.33	5.27
Spouse’s age (years) (min. 20–max. 46)		32.86	5.83
Duration of marriage (years) (min. 1–max. 30)		6.04	5.03
Number of pregnancies (min. 1–max. 6)		2.10	1.13
Number of children (min. 0–max. 5)		0.97	0.99
		**n**	**%**
Educational status	Primary education	32	11.4
	Secondary education	96	34.3
	Higher education	152	54.3
Spouse’s educational status	Primary education	21	7.5
	Secondary education	100	35.7
	Higher education	159	56.8
Employment status	Employed	53	18.9
	Unemployed	227	81.1
Family Type	Nuclear	247	88.2
	Extended	33	11.8
Pregnancy planning	Planned	227	81.1
	Unplanned	53	18.9
Type of pregnancy	Spontaneous	262	93.6
	Assisted reproductive techniques	18	6.4
Pregnancy-related discomfort/disorder	Yes	34	12.1
	No	246	87.9
Regular prenatal care	Receiving	145	51.8
	Not receiving	135	48.2
Regularly following pregnancy health info sources	Following	187	66.8
	Partially following	87	31.1
	Not following	6	2.1

SD: Standard Deviation; n: number; %: percentage.

**Table 2:** Distribution of mean scores for the Maternal Health Literacy Scale in Pregnancy (MHLS-P), and the Healthy Lifestyle Behaviors Scale in Pregnant Women (HLBS-PW).

Scales	X ± SD	Min.	Max.
**MHLS-P Total**	77.29 ± 14.85	20.31	100.00
Maternal health information subscale	75.76 ± 18.16	10.71	100.00
Making research for maternal health information subscale	71.48 ± 18.99	8.33	100.00
Evaluating maternal health information subscale	77.70 ± 18.30	8.33	100.00
Taking decision related to the maternal health and maternal health behavior subscale	81.60 ± 14.60	18.33	100.00
**HLBS-PW Total**	118.21 ± 14.87	51.00	145.00
Pregnancy responsibility subscale	17.79 ± 2.56	4.00	20.00
Nutrition subscale	17.95 ± 2.41	6.00	20.00
Hygiene subscale	33.90 ± 6.11	19.00	45.00
Physical activity subscale	10.74 ± 2.78	3.00	15.00
Travel subscale	20.63 ± 4.27	5.00	25.00
Acceptance of pregnancy subscale	17.17 ± 2.82	4.00	20.00

X ± SD: Mean ± standard deviation; min.: Minimum; max.: Maximum.

**Table 3:** Comparison of Healthy Lifestyle Behaviors Scale in Pregnant Women (HLBS-PW) Mean Scores According to Descriptive Characteristics.

Characteristics		HLBS-PW	Test; *p*
		X ± SD	
Educational status	Primary education	111.09 ± 20.45 ^a^	F = 8.331
	Secondary education	115.87 ± 15.45 ^a^	0.000
	Higher education	121.19 ± 12.21 ^b^	
Spouse’s educational status	Primary education	116.66 ± 18.22 ^ab^	F = 5.970
	Secondary education	114.43 ± 16.08 ^a^	0.003
	Higher education	120.80 ± 13.04 ^b^	
Employment status	Employed	122.16 ± 13.20	t = 2.163
	Unemployed	117.29 ± 15.11	0.031
Family Type	Nuclear	118.98 ± 13.88	t = 1.802
	Extended	112.45 ± 20.19	0.080
Pregnancy planning	Planned	119.00 ± 13.53	t = 1.488
	Unplanned	114.83 ± 19.37	0.142
Type of pregnancy	Spontaneous	118.26 ± 15.10	t = 0.195
	Assisted reproductive techniques	117.55 ± 11.14	0.846
Pregnancy-related discomfort/disorder	Yes	113.91 ± 13.75	t = −1.809
	No	118.81 ± 15.53	0.072
Regular prenatal care	Receiving	120.86 ± 13.75	t = 3.132
	Not receiving	115.37 ± 15.53	0.002
Regularly following pregnancy health info sources	Following	121.81 ± 12.72 ^a^	F = 23.502
	Partially following	112.10 ± 15.17 ^b^	0.000
	Not following	94.83 ± 23.80 ^c^	
		**r**	***p***
Age (years)		0.030	0.614
Spouse’s age (years)		−0.046	0.443
Duration of marriage (years)		−0.041	0.492
Number of pregnancies		−0.036	0.546
Number of children		−0.047	0.438

X ± SD: mean ± standard deviation; t: Independent samples *t* test; F: One-way analysis of variance (ANOVA). ^a,b,c^: Representation of differences according to the Tukey test (indicates a significant difference between groups with different letters).

**Table 4:** Correlation values between HLBS-PW, MHLS-P, and subscale scores of women.

Scales *		MHLS-P Total	Maternal Health Information	Making Research for Maternal Health Information	Evaluating Maternal Health Information	Taking Decision Related to the Maternal Health and Maternal Health Behavior
HLBS-PW total	r **	0.683	0.596	0.490	0.549	0.652
	*p*	0.000	0.000	0.000	0.000	0.000
Pregnancy responsibility	r **	0.485	0.407	0.296	0.387	0.522
	*p*	0.000	0.000	0.000	0.000	0.000
Hygiene	r **	0.492	0.436	0.262	0.380	0.534
	*p*	0.000	0.000	0.000	0.000	0.000
Nutrition	r **	0.502	0.466	0.381	0.410	0.418
	*p*	0.000	0.000	0.000	0.000	0.000
Physical activity	r **	0.474	0.419	0.434	0.361	0.407
	*p*	0.000	0.000	0.000	0.000	0.000
Travel	r **	0.477	0.386	0.328	0.414	0.502
	*p*	0.000	0.000	0.000	0.000	0.000
Acceptance of pregnancy	r **	0.455	0.392	0.371	0.343	0.435
	*p*	0.000	0.000	0.000	0.000	0.000

* Pearson correlation analysis; ** Correlation coefficient (r = 0.00–0.25 very weak, r = 0.26–0.49 weak, r = 0.50–0.69 moderate, r = 0.70–0.89 high, r = 0.90–1.00 very high).

**Table 5:** Regression analysis of the effect of maternal health literacy on healthy lifestyle behaviors in pregnant women.

Independent Variables	Beta	Standard Error	Standard Beta	T	*p*	95% Confidence Interval Minimum Maximum	
Constant	65.386	7.452	-	8.775	0.000	50.716	80.056
Maternal Health Literacy Scale in Pregnancy scale total score	0.636	0.052	0.635	12.192	0.000	0.533	0.739
Educational status	1.420	1.188	0.066	1.196	0.233	−0.918	3.758
Employment status	1.116	1.777	0.029	0.628	0.531	−2.383	4.615
Spouse’s educational status	0.485	1.246	0.021	0.389	0.697	−1.968	2.939
Receiving regular prenatal care	−0.484	1.353	−0.016	−0.358	0.721	−3.148	2.180
Regular monitoring of pregnancy-related health information sources	−1.704	1.435	−0.060	−1.187	0.236	−4.530	1.122

Multiple Linear Regression Analysis. Dependent variable: Healthy Lifestyle Behaviors Scale total score. Model: R = 0.689; R^2^ = 0.474; F = 41.081; *p* < 0.001; Durbin-Watson = 1.904.

## Data Availability

The data underlying this article are not publicly available due to ethical and privacy restrictions. The participants of this study did not give written consent for their data to be shared publicly, as the dataset contains sensitive personal health information collected from pregnant women.

## Abbreviations

The following abbreviations are used in this manuscript:

HL	Health literacy
MHLS-P	Maternal Health Literacy Scale in Pregnancy
HLBS-PW	Healthy Lifestyle Behaviors Scale in Pregnant Women
AMA	American Medical Association
HLBS	Healthy Lifestyle Behaviors Scale
